# Evaluating the communities care program: best practice for rigorous research to evaluate gender based violence prevention and response programs in humanitarian settings

**DOI:** 10.1186/s13031-018-0138-0

**Published:** 2018-01-31

**Authors:** N. Glass, N. Perrin, A. Clough, A. Desgroppes, F. N. Kaburu, J. Melton, A. Rink, S. Read-Hamilton, M. Marsh

**Affiliations:** 1 Johns Hopkins University School of Nursing, 525 North Wolfe Street, Unit 435, Baltimore, MD 21205 USA; 2 Comitato Internazionale per lo Sviluppo dei Popoli (CISP) Somalia, Nairobi, Kenya; 3 UNICEF, Juba, South Sudan; 4 Consultant, Gender based violence in Emergencies, Sydney, Australia; 5 UNICEF, New York, NY USA

## Abstract

**Background:**

Gender-based violence (GBV) is a significant issue for women and girls in humanitarian settings. Innovative primary prevention programs are being developed and implemented with existing response programs to change harmful social norms that sustain GBV in humanitarian settings. Social norms are expectations of how women, men, girls and boys should behave, who should have power and control over behavior, and how families and communities value women and girls and support their rights and opportunities.

**Methods:**

The United Nations Children’s Fund (UNICEF) led Communities Care program is a primary prevention and response program designed from the understanding that within the context of conflict and displacement, there is an opportunity for positive change in social norms that support gender equity, and decrease GBV. The goal is to support communities in humanitarian settings to create healthy, safe and peaceful environments with quality response services for women and girls by transforming harmful social norms that uphold violence into norms that promote dignity, equity, and non-violence.

**Conclusion:**

This manuscript will highlight the use of best practices in GBV research to rigorously evaluate the Communities Care program in two diverse in humanitarian settings, Somalia and South Sudan.

## Introduction

Gender-based violence (GBV) remains one of the most prevalent and persistent issues facing women and girls globally [1–4]. War and other humanitarian disasters place civilians at increased risk of many forms of violence [5–7]. The fear of violence can promote forced and mass displacement. Women and girl refugees and internally displaced persons (IDPs), as well as host community members, are exposed to multiple traumatic experiences, including GBV [8–10]. The Interagency Standing Committee (IASC) 2015 *Guidelines for Integrating GBV Interventions in Humanitarian Action* [11] defines GBV as an umbrella term for any harmful act that is perpetrated against a person’s will and that is based on socially ascribed (i.e. gender) differences between males and females. It includes acts that inflict physical, sexual or mental harm or suffering, threats of such acts, coercion, and other deprivations of liberty. Importantly, these harmful acts can occur in public and in private. In a humanitarian context, GBV may occur during the conflict, during displacement and settlement in new communities and countries, and in the home by an intimate partner or other family member [5].

While GBV is not unique to humanitarian emergencies, women and girls are vulnerable to violence across the trajectory of conflict and displacement [12–17]. Several individual, family and structural factors have been cited to increase women and girls’ risk of GBV in humanitarian settings including extreme poverty, minority status, social norms that limit women/girls movement and access to all resources necessary for survival (e.g., fuel, shelter, food and water, etc.) and disrupted family and community support systems, among others [8, 17–21]. GBV has significant short and long-term consequences for the safety, health and functioning of women and girls [22–25]. Often these negative consequences are never addressed because women and girls do not disclose violence to authorities or access existing health and social services. Women and girls report non-disclosure is often related to harmful social norms that blame the victim for the assault, prioritize family honor and dignity over the health and safety needs of the survivor, and acceptance of sexual violence and other forms of GBV as normal and expected [10, 16, 26, 27]. Given the significant experience and risk of sexual violence and other forms of GBV faced by women and girls in conflict and other humanitarian settings, global and local organizations are collaborating to develop, implement and evaluate innovative GBV primary prevention and response interventions in diverse humanitarian settings [15, 17, 28, 29].

Importantly, as international and local organizations are implementing GBV prevention and response programs, there is a critical need for guidance and recommendations on best practices for conducting research to rigorously evaluate these programs [30].

## Background

Collaborations in global humanitarian settings have advanced from programs that focus solely on the multi-sectoral response to GBV to include primary prevention activities, which aim to prevent GBV [15, 17, 28, 29]. Primary prevention strategies target transforming conditions that support GBV, promoting positive behaviors and developing skills to model behaviors so others are able to adopt new norms and behaviors in order to prevent GBV [15]. Communities have shared beliefs and unspoken rules that can send the message that GBV against girls and women is acceptable, even normal [15]. Norms are expectations of how women, men, girls and boys should behave, who should have power and control over behavior, and how families and communities value women and girls and support their rights and opportunities [15]. Local institutions and providers of services, such as health care, education and justice services can reinforce harmful norms by, for example, denying that sexual harassment and violence exists in the community, by blaming women and girls for the sexual assault when they seek care, and by police and the legal system refusing to hold a husband accountable for abusing his wife. It is also true that in humanitarian settings there are often resources and programs available that may not be present in non-humanitarian settings that can be leveraged to change harmful community and institutional norms to norms that promote the health and safety of women and girls. Specifically, in these diverse and complex settings, there are global and local organizations that are working to increase community and institutional awareness of GBV as a violation of human rights and increasing access to quality GBV services through multiple sectors, health care, education, protection and justice [17]. Further, given the changes in family and community structures (e.g., absence of male family member(s) and/or the necessity of women to work in non-traditional roles outside the home) there can be a shift in economic control and decision-making that leads to awareness by community members of a shift in power relations and the presence of harmful social norms and increase the opportunity for community dialogues to diagnose norms that maintain inequity and GBV and identify actions by the community to change these norms [15]. Acknowledging the potential of the humanitarian setting as an opportunity to integrate primary prevention programming with response programs and building on their previous experience in social norms change in female genital mutilation/cutting [31], the United Nations Children’s Fund (UNICEF) developed the Communities Care program. Communities Care is a social transformation program to end violence against women and girls in conflict-affected communities [32].

### Overview of UNICEF’s communities care program

As noted above, the Communities Care program was developed from the understanding that within the context of conflict and displacement, there is an opportunity for positive change in social norms that support gender equity, and decrease GBV. The ultimate goal of the program is to support communities in leading in the creation of healthy, safe and peaceful environments with quality response services for women and girls by transforming harmful social norms that uphold violence against women and girls into norms that promote dignity, equality, and non-violence [15, 32]. As the manuscript focuses on best practices for conducting research to rigorously evaluate primary prevention and response programs in humanitarian settings, only a brief overview of the program and objectives are presented below. A detailed description of the program is published elsewhere [15].

The Communities Care program prioritizes partnerships with local communities in humanitarian settings to respond to the urgent need to increase access to quality health care and support services for survivors of GBV, as well as develop and test effective strategies to prevent GBV in conflict-affected communities. The program has two objectives that are implemented through separate but interrelated strategies: 1) to increase the quality, access, and coordination of compassionate care and support of women and girls who experience GBV in conflict-affected settings by strengthening community-based response services across diverse sectors (e.g., health, psychosocial, protection and education); and 2) to change social norms that maintain and tolerate GBV and catalyze community led prevention actions [15].

Communities Care is a theory-driven program. A feminist-informed public health approach to GBV prevention and response is integrated within the ecological framework. The ecological framework acknowledges the need to comprehensively address the multiple and interacting levels (e.g., individual, family, community, institution and social) of factors that maintain and tolerate GBV. Communities Care also builds on the theory of change based on UNICEF led research suggesting that for harmful norms to be abandoned in a community, there must be a shift in wider social expectations about behavior as well as personal beliefs of community members [15]. The program’s pathway of social norms change includes actions (e.g., partnership, capacity-building, resourcing and mentorship) to strengthen community-based response across diverse response sectors (e.g., education, health, psychosocial and protection) for women and girls who experience GBV [15]. The next step in the pathway is to concentrate on engaging diverse and influential community members in structured dialogues that aim to lead to collective reflection and exploration on values, aspirations, and existing social norms that tolerate GBV, and alternatives to replace harmful social norms. The Communities Care curriculum on social norms change is delivered through 15-weeks of structured and facilitated dialogues led by trained community members. Single and mixed sex groups of adults and adolescents are brought together to build awareness and consciousness about shared values of respect for human dignity, fairness, and justice; to connect their experiences of violence and injustice to the experiences of others; and to analyze how social norms contribute to GBV. The goal of the facilitated dialogues is to empower diverse and influential community members to work together to diagnose GBV as a problem in the community, identify norms that sustain norms that support gender equity, safety and well-being [15].

This manuscript will detail the design, methods and limitations of the comprehensive evaluation of a novel United Nations Children’s Fund (UNICEF) GBV primary prevention and response program implemented in Somalia and South Sudan. The paper will highlight the use of best practices recommended to rigorously evaluate the Communities Care program in two diverse humanitarian settings, Somalia and South Sudan. The best practices include: 1) establishment of local partnerships; 2) training and capacity-building with global and local teams; 3) using formative research methods to define and diagnose social norms; 4) use of mixed methods (qualitative and quantitative methodologies) to develop and test measures of social norms; 5) contextualizing the program to diverse settings; and 6) use of qualitative and quantitative methods/data to longitudinally evaluate the impact of the program on change in norms that sustain sexual violence and other forms of GBV.

## Methods

The research design and methods to evaluate the Communities Care program was implemented in three phases: 1) Inception Phase, which includes the development of the in-country partnerships to define and diagnose social norms in the target settings; 2) Measurement Phase, which uses formative methods to develop and evaluate the psychometric properties of a social norms measure to be implemented in the impact evaluation phase; 3) Impact evaluation phase, a longitudinal study to determine the effectiveness of Communities Care program on changes in social norms within targeted communities in Somalia and South Sudan.

### Settings

The countries of Somalia and South Sudan are characterized by ongoing instability, violent conflict and high levels of displacement. In South Sudan, more than 2.3 million people have been forced to flee their homes since the conflict began in 2013, including 1.66 million IDPs [33]. In Somalia, about 4.9 million people are in need of life-saving and livelihoods support and 1.1 million remain internally displaced [34]. In South Sudan, two communities in Yei County in Central Equatoria State and Gogrial West County in Warrap State are participating in the Communities Care program. In Southern and Central Somalia, two districts of Mogadishu, Yaqshid and Bondhere are participating. These communities were selected based on a number of criteria, including prevalence of GBV and sexual violence, safe access, and security for participants and staff to conduct the project and establish relationships with national and regional governmental authorities and ministries. Also critical to implementation was the availability of international and/or local non-governmental organizations (NGO) with capacity to provide GBV services in the targeted communities, and an interest and willingness of local leadership and authorities to host the implementation and research to evaluate the Communities Care program. Importantly, when we started the program in both countries (2012), the targeted communities were selected because they were considered secure and calm. In the two counties in South Sudan participants were primarily residents of the communities as both Central Equatoria and Warrap State had been relatively free of armed conflict, however, for example, since July 2016, Yei County in Central Equatoria has experienced significant violence, insecurity and mass displacement of civilians into neighboring countries of Democratic Republic of Congo and Uganda. In Mogadishu insecurity is a daily experience and is related to ongoing attacks of civilians and African Union peacekeepers by Al-Shabaab militants. The ongoing violence and insecurity continues to impact both established residents and internally displaced people living in informal settlements within the two districts participating in the Communities Care program. Throughout the implementation and evaluation of Communities Care, security in Southern and Central, Somalia and Central Equatoria and Warrup State, South Sudan has been dynamic resulting in all the participating communities experiencing periods of insecurity, including an exodus of civilians from some communities and an increase in displaced persons moving into informal settlements in other participating communities for safety.

## Phase one: inception phase

In phase one, the collaborators from UNICEF-Headquarters (HQ), Somalia and South Sudan in-country UNICEF offices, and Johns Hopkins University work closely with established international and local NGOs to build partnerships to successfully define and diagnose harmful social norms and establish relationships with diverse services for GBV survivors. In South Sudan, two national NGOs, Voice for Change in Central Equatoria State and The Organization for Children Harmony in Warrup State and an Italian NGO, Comitato Internazionale per lo Sviluppo dei Popoli (CISP) working in Mogadishu and other regions of Somalia joined the project with roles in the Communities Care program implementation and evaluation. NGOs working in the targeted communities have multiple challenges common to other humanitarian settings. These challenges are insecurity, lack of access to residents in the communities, especially during the rainy seasons when roads become impassable, and the limited availability of a skilled workforce to deliver services and monitor assess to and quality of these services. These challenges can be overcome with dedicated resources and regular communication between partners, with participatory planning and capacity building for all partners throughout the program.

### Mapping GBV services and readiness to respond to survivors

The Communities Care program uses a participatory approach thus engaging local partners in leading the mapping and assessing readiness of diverse sectors (e.g., legal, health, psychosocial and education) to provide safe and confidential services to GBV survivors. Prior to implementing Communities Care, a workforce available and willing to provide quality services to GBV survivors is essential. In the targeted settings, the local NGO implementing partners mapped GBV services throughout the project implementation as ongoing service mapping is critical because the humanitarian context is dynamic and over time services close as funding ends as well as new programs and services are created. An example of a readiness assessment of services to respond to GBV survivors may be working with a local primary health clinic in the targeted community to determine if clinical protocols for survivors of sexual violence and other forms of GBV exist and if nurses and midwives working in the clinic have received training on the clinical protocols. Further, the clinical protocols can be reviewed and the providers interviewed to determine if a referral pathway to community-based services including psychosocial support, education, protection and justice services are available. Mapping the services and assessing readiness supports the local partners’ ability to provide capacity building across diverse service sectors on harmful social norms while providing quality clinical and community-based services that do not reinforce harmful norms, such as blaming the victim, refusing treatment and/or refusing to provide appropriate referrals to survivors.

### Building partner staff capacity to conduct formative research

For the formative research of phase one, the collaborators implemented a three-day training that included both classroom and field activities to prepare local partner staff to safely and confidentially conduct focus group discussions and individual interviews with key stakeholders on harmful social norms and GBV response services. As formative research engages human subjects participation, it is the responsibility of the collaborating organization to require partner staff to successfully demonstrate understanding of ethical principles in research. Prior to our in-person research training, all local partner staff complete an on-line course on Research Ethics using the FHI360 Training Curriculum https://www.fhi360.org/sites/all/libraries/webpages/fhi-retc2/. The training included a review and discussion of ethical principles of informed consent, confidentiality, voluntary participation, benefits and risks of participation and safety concerns. The next component of the research training includes NGO partner staff actively defining, diagnosing and examining their personal beliefs and behaviors associated with social norms in their community. Importantly, all partner NGO staff participating in the formative research had previously received extensive training on GBV prevalence, risk factors for GBV and diverse services as well as the referral pathways in the humanitarian setting. Formative research methods for focus group discussions and individual interviews used an established script with probes and scenarios to engage participants in discussions about harmful norms and GBV response services in communities. The training also covers building rapport and respect for focus group and interview participants, including active listening, probing for follow-up information, and keeping control of a group and handling difficult situations, such as conflict between participants. Training participants were asked to volunteer to role-play scenarios to demonstrate successful facilitation and management of group dynamics with different communication techniques. Additionally, local partner staff reviewed and discussed the focus group/interview questions and suggested changes as appropriate for the context and language. Participant distress or need for immediate GBV services during a group/interview and a protocol for referring participants to appropriate services was developed during the training using the findings from local partners’ mapping and readiness activities. The concept of vicarious trauma (e.g., provider trauma associated with hearing and providing services to survivors and other traumatized groups) was discussed with partner staff, and a system for debriefing and support was developed within the workflow of the Communities Care program. The protocol for recruiting focus group members from the target community was reviewed and finalized. The training also included mock recruitment and focus group activity that provided implementing partner staff with an opportunity to practice what they had learned during the training. The facilitators and participants in the mock focus group were instructed on scenarios to be “difficult” in the group as to provide an opportunity for facilitators to practice and demonstrate how to handle challenges during the groups. After each mock group, there was an opportunity to debrief and discuss and make modifications for focus group/interview protocols as recommended by implementing partners prior to finalizing the protocol for implementation.

### Translation and transcription

As a component of capacity building, with guidance by our local partners, all focus group materials were translated and back-translated and piloted with community members to insure understandability and appropriate terminology. Further, if consent is obtained, the focus groups and interviews are audio-recorded and transcripts prepared in the local language and then translated to English for use by all the team members. Transcripts in both the local language and English allowed for all members of the team to review to enhance understanding and resolve questions in meaning when collaborating on diagnosing social norms in diverse community settings.

### Engaging key stakeholders in examining and diagnosing social norms

The partners collaborated across diverse sectors and community groups to identify key stakeholders (e.g., religious leaders, traditional and administrative authorities, teachers, health care providers, GBV and human rights advocates, women’s group leaders, business leaders, etc.) that have influence on beliefs, behaviors and actions in the targeted communities. The key stakeholders were invited to participate in the focus groups and individual interviews to diagnose social norms that sustain sexual violence and other forms of GBV. The discussions with the stakeholders defined and diagnosed: a) norms that sustain GBV in households and the community; b) content/topics to stimulate critical reflection for changing social norms that sustain GBV in households and the community; c) women/girls role in education, leadership and peace building in the community; and d) barriers (bottlenecks) associated with household and community acceptance and silence about disclosure of GBV to authorities.

### Analysis and social norms measure development

As noted above, the focus group and interview transcripts were translated to English to allow for all partners to participate in the analysis. An iterative, analytic approach was followed using Crabtree and Miller’s five-step approach to qualitative interpretation: 1) describing, 2) organizing, 3) connecting, 4) corroborating, and 5) representing. Regular debriefing meetings were held with all partners to identify preliminary findings and facilitate an iterative data collection and analysis [35]. An initial set of codes were developed based on core domains from the discussions and interviews: a) social norms that sustain GBV; b) women/girls role in education, leadership and peace building in the community; and c) barriers (bottlenecks) associated with household and community acceptance and silence about disclosure of GBV. These codes were then used by the team to write questions for a social norms measure to be implemented with community members in both countries in phase two.

## Phase 2: measurement phase

Measuring social norms is relatively new and therefore as a component of the evaluation, the partners needed to conduct a measurement phase to develop a social norms instrument that was reliable and valid in measuring changes in harmful social norms in communities implementing the Communities Care program. To our knowledge, there were no social norms measures that addressed GBV that had been previously developed and tested in humanitarian settings. The lack of social norms measures is likely associated with the multiple theoretical and disciplinary perspectives and understandings of social norms, including how norms differ from personal beliefs, opinions, attitudes, and behavior [15, 36]. As noted by Heise and Monji [37], a norm is a social construct and is a collectively shared belief about what others do (what is typical) and what is expected of what others do within the reference group (what is appropriate). Efforts to measure social norms related to GBV have resulted in information on beliefs and attitudes rather than social norms. For example, an attitude is an individually held belief and has an evaluative component, suggesting that something is right or wrong, “A good wife should have sex with her husband even if she does not want to.” To measure changes in social norms, the norms that sustain GBV must first be diagnosed in collaboration with key stakeholders in targeted communities (see phase one). Diagnosing the social norm involves identifying and examining the multiple and inter-related factors (e.g., structural, family, individual) that maintain a harmful behavior. At the end of phase one, we had collaboratively diagnosed the presence and strength of a norm, and the behaviors that reflect the norm and used this diagnosis to develop a social norms measure to be evaluated in phase two. Importantly, to measure social norms, a person must be asked about a behavior from multiple perspectives, including personal beliefs regarding the behavior, beliefs about how influential others expect one to behave, and beliefs about how others in the community behave [15, 31, 37]. A reliable and valid measure of norms and completing a psychometric analysis of the measure with community members in the targeted settings was an essential component to be able to conduct an impact evaluation to examine the effectiveness of the Communities Care program on change in certain harmful norms in diverse humanitarian settings.

### Measuring social norms

As indicated above, the social norms measure focused on multiple perspectives. For example, to measure how influential others (e.g., mothers, fathers, other family members, peers, religious leaders, teachers, governmental authorities) expect one to behave, we first asked participants to identify influential others whose opinions matter to them related to GBV. Once the influential others were identified, the research assistants (RAs) read the statement to community participants: *“thinking about those people whose opinion matters to you, how many of these influential people would blame women/girls if they are raped.”* The responses are on a Likert-scale that ranges from none of them to all of them. The full social norms measure will be published once the Communities Care program evaluation is complete in 2017.

The measurement phase included a one time quantitative survey using the social norms measure. As in phase one, local implementing partners participated in training and led the protocol development for inviting community members to participate, building rapport and trust with participants, obtaining informed consent, addressing and minimizing refusals and documentation. The social norms measure was reviewed item by item and discussed to ensure all were in agreement on the wording and meaning of each question. Importantly, during the training, facilitators reviewed with RAs the potential for vicarious trauma and tips for recognizing and managing distress.

### Sampling and randomization for phase two

In each community within the two countries, trained local RAs recruited and consented 200 community members of varying age and sex to complete the survey with the social norms measure. Each RA was responsible to recruit and administer the survey to a total of 20 community participants across the sex and age categories. As suggested by the in-country teams, male RAs recruited male participants and female RAs recruited female participants. Each RA recruited participants in all age groups.

### Collaborating to identify participants

The local partners had established relationships with community leaders, but prior to implementing the survey the partners assigned to visit the community authorities and remind them of the project asked the leaders to designate one “community guide” for each RA. The community guides helped the RAs to safely move and find their way around the community as well as introduce the RA and engage in discussions with potential participants. Once a community member agreed to participate, the community guides would not be present during the consent process and administration of the survey.

### Phase two field procedures

To cover the community that each RA had been assigned, he/she started from a central point determined with the community guide and knocked on the door of every third house/dwelling. In counting the third house/dwelling, the RAs were asked to count the houses on both sides of the street/pathway. If nobody was home or the person they met at the house/dwelling was not willing to participate or did not match the sampling target for sex/age, the RA went to the next house/dwelling. Once a participant who met the targeted sex/age was identified and agreed to participate in a household, the RA worked with the participant to find a private and comfortable place to provide informed consent and administer the survey. A survey was completed with only one eligible participant in each household. The RA provided each participant with informed consent information using the script provided on the iPad that was approved by the appropriate federal and state government ministry in each country and the Johns Hopkins Medical Institution Institutional Review Board (IRB). The government ministry provided a letter of approval to Johns Hopkins and the local implementing partners to use as they reached out to authorities and key stakeholders to implement the research and evaluation. The consent process consists of explaining the purpose of the survey, how long it would take (up to 30 min), confidentiality, and the voluntary nature of participation. If the participant provided verbal consent the RA administered the survey and recorded their responses on the iPad. At the end of the survey, RAs thanked the participant for their time and answered any questions prior to moving on. The RA then started the process to identify the next eligible participant by going to the next third house/dwelling on the street/pathway. RAs returned to the partner office at the end of the data collection day to sign-in the iPads and turn in tracking sheets to the supervisor. The supervisor connected all the iPads to the wireless Internet in the office and uploaded the data to the secure server hosted by Hopkins and documented the number of surveys completed in the sampling frame to monitor progress. Once the data was uploaded, it was automatically removed from the iPads.

### Psychometric analysis

A psychometric analysis of the social norms measure was conducted in order to avoid moving forward with the Communities Care program impact evaluation (Phase 3) using a measure that is biased. As part of the protocol, the team focused on the reliability (e.g., extent to which the measure is consistent) and validity (e.g., accuracy of the measure) of the items and measure using diverse analytical techniques. For phase two, the collaborators used factor analysis, internal consistencies reliability, and tests of hypothesized differences across groups (e.g., sex, age). The analyses were used to refine the measure and establish the psychometric properties prior to conducting phase three. The social norms measure will be published at completion of phase three, the impact evaluation.

## Phase three: impact evaluation

As noted previously, rigorous studies that examine the effectiveness of GBV prevention and response interventions are limited. For phase three, the collaborators designed a longitudinal, mixed-methods community-based trial with participating communities in both countries randomized to either intervention or control communities. The impact evaluation is ongoing and the aims are: 1) to determine the effectiveness of the Communities Care program (intervention) on harmful social norms that sustain GBV against women and girls in each participating country setting*.* The team hypothesized that communities participating in the intervention will report a decrease in harmful social norms in comparison to control communities; and 2) to determine the effectiveness of the Communities Care program on response from diverse sectors (e.g., health, education, protection, and justice) to GBV survivors. The team hypothesized that participants in the intervention communities will report improved service response to GBV survivors compared to control communities.

### Longitudinal research training

In phase three, the team was able to build on the existing local implementing partner expertise in recruitment, interview skills, and human subjects protection to conduct the longitudinal study. The team used a Train-the-Trainer (TOT) approach at all sites, reducing the cost associated with having external trainers. Unique to this phase was the importance of community participant retention over the planned one-year follow-up. The local team developed a protocol that required asking participants for safe contact information including a cell phone number to send SMS reminders of upcoming follow-up interviews as well as provided incentives to reimburse the expertise and time of participants.

### Participant eligibility, recruitment and retention for longitudinal research

To achieve the aims, adult men and women (*n* = 200, 15 years and older and head of household) residents in intervention and control communities are eligible to participate in three separate interviews (baseline, midline and endline) over approximately 12 months. Additional to the community sample, the team is interviewing service providers, participants in the Communities Care 15-week community dialogues and women/girls that access health services to examine changes in social norms and satisfaction with services in both intervention and control communities.

### Evaluation measure

The study measure consists of demographic questions, the social norms measure and other validated measures related to GBV services and satisfaction with care used previously by the team and others in similar low-resource and conflict-affected settings. Prior to official recruitment and survey administration, 10 adult men and women pre-testers in each setting complete the evaluation process with RAs. The pre-testers are asked to evaluate the survey questions, translation, content and understandability of the questions. If the RAs report challenges with the survey questions, the survey is modified and retested in collaboration with the local partners. The final approved survey is translated and back-translated using the process outlined previously. As in phase two, the study survey is programmed into secure iPads.

### Data collection and analysis for impact evaluation

The baseline data collection in the intervention (Communities Care program) and delayed control communities was initiated after recruitment and randomization but prior to implementation of the Communities Care program. All research related activities are completed in a private setting of the participant’s choice, most often in their home. The study survey is administered only after informed oral consent is obtained with the eligible adult male or female participant. All participants are informed that their refusal to participate or stop/withdraw from the study would not affect their access to services or programs delivered in the community. Trained RAs use the iPad to conduct the survey and the use of iPads for data collection is beneficial in multiple ways: (1) reduced logistical burden of printing and managing the large number of paper surveys; and (2) real-time access to the data to monitor data quality and identification of issues so that they could be remedied with supervisors and staff. Additionally, in our previous research, participants expressed confidence and comfort when answering questions with the use of the iPad as compared to paper-based surveys where the RAs are writing down responses. Data recorded on iPads are encrypted; once uploaded to a central US-based server, the data are automatically erased from the iPad for security. The primary outcome of the impact evaluation is a positive change in social norms that sustain GBV at endline in the Communities Care program communities compared to delayed control communities in both South Sudan and Somalia. All analyses are based on intent-to-treat principles. Multilevel modeling will be used to test the effectiveness of the intervention communities compared to delayed control communities on changing harmful social norms (Aim 1). Time (baseline, midline, endline) will form the first level of the model and participant the second level of the model. Generalized Estimating Equations (GEE) will be used to test Aim 2 to determine the effectiveness of the Communities Care program on GBV response services in intervention communities compared to delayed control communities. GEE was selected because all women/girls utilizing services during a selected period of time will be invited to participate. It is possible that some women will participate at more than one time point. GEE can incorporate this dependency into the model, which tests for differences in changes over time between the intervention and delayed control communities on the outcome of social norms change.

## Discussion and conclusion

Our comprehensive evaluation of the Communities Care program, a GBV prevention and response program, is guided by published recommendations for best practices in GBV research in humanitarian settings. We provide details on applying recommended best practices throughout the three phases of the evaluation. Specifically, establishing partnerships with local and global partners and collaboratively building in-country capacity to successfully conduct formative research to diagnose harmful social norms associated with GBV and develop a measure of these harmful norms within a challenging humanitarian context. Given the humanitarian context, the diagnosis recognized that previous norms and behaviors are being disrupted because of the multiple changes in structural, family and individual factors, such as the loss of economic opportunities for men requiring women to work outside the home and taking on the role of financially supporting a family. We also detail our collaboration to test a reliable and valid social norms measure to represent multiple perspectives, personal beliefs about the norms, beliefs about how influential others expect one to behave and beliefs about how others in the community behave related to GBV. The social norms measure is used as the primary outcome of social norms change in the ongoing longitudinal impact evaluation to examine the effectiveness of the Communities Care program in two humanitarian emergencies, Somalia and South Sudan. The paper is useful for global and local stakeholders (e.g., governments, donors, practitioners) that are invested in implementing and evaluating GBV prevention and response programs in humanitarian settings, but have limited technical resources to guide stakeholders in best practices in GBV research.

## References

[1] Decker MR (2015). Gender-based violence against adolescent and young adult women in low- and middle-income countries. J Adolesc Health.

[2] Devries KM (2013). Global health. The global prevalence of intimate partner violence against women. Science.

[3] Watts C, Zimmerman C (2002). Violence against women: global scope and magnitude. Lancet.

[4] Garcia-Moreno C (2006). Prevalence of intimate partner violence: findings from the WHO multi-country study on women's health and domestic violence. Lancet.

[5] Vu A, Adam A, Wirtz A, Pham K, Rubenstein L, Glass N, Beyrer C, Singh S. The Prevalence of Sexual Violence among Female Refugees in Complex Humanitarian Emergencies: a Systematic Review and Meta-analysis. PLOS Currents Disasters. 2014 Mar 18. Edition 1. 10.1371/currents.dis.835f10778fd80ae031aac12d3b533ca7. 10.1371/currents.dis.835f10778fd80ae031aac12d3b533ca7 PMC4012695 24818066

[6] Wirtz AL (2014). Gender-based violence in conflict and displacement: qualitative findings from displaced women in Colombia. Confl Health.

[7] Sloand E (2015). Barriers and facilitators to engaging communities in gender-based violence prevention following a natural disaster. J Health Care Poor Underserved.

[8] Rubenstein BL, Stark L. The impact of humanitarian emergencies on the prevalence of violence against children: an evidence-based ecological framework. Psychol Health Med. 2017;22(sup1):58–66. 10.1080/13548506.2016.1271949 28064522

[9] Stark L, Landis D (2016). Violence against children in humanitarian settings: a literature review of population-based approaches. Soc Sci Med.

[10] Wirtz AL (2016). Comprehensive development and testing of the ASIST-GBV, a screening tool for responding to gender-based violence among women in humanitarian settings. Confl Health.

[11] Committee I.-A.S (2015). Guidelines for integrating gender-based violence interventions in humanitarian action: reducing risk, promoting resilience and aiding recovery.

[12] Hynes M (2004). A determination of the prevalence of gender-based violence among conflict-affected populations in East Timor. Disasters.

[13] Wirtz A, et al. Development of a screening tool to identify female survivors of gender-based violence in a humanitarian setting: qualitative evidence base from research among refugees in Ethiopia. Confl Heal. 2013;7(1):13. 10.1186/1752-1505-7-13 PMC3695841 23758886

[14] Wirtz A, et al. Gender-based violence in conflict and displacement: Qualitative findings from displaced women in Colombia. Confl Health. 2014;8:10. https://doi.org/10.1186/1752-1505-8-10. 10.1186/1752-1505-8-10 PMC4115473 25076981

[15] Marsh SR-HM (2016). The communities care programme: changing social norms to end violence against women and girlsin conflict-affected communities. Gend Dev.

[16] Stark L (2013). Measuring the incidence and reporting of violence against women and girls in liberia using the ‘neighborhood method’. Confl Health.

[17] Tappis H, Freeman J, Glass N, Doocy S. Effectiveness of Interventions, Programs and Strategies for Gender-based Violence Prevention in Refugee Populations: An Integrative Review. PLOS Currents Disasters. 2016 Apr 19 . Edition 1. 10.1371/currents.dis.3a465b66f9327676d61eb8120eaa5499. 10.1371/currents.dis.3a465b66f9327676d61eb8120eaa5499 PMC4865365 27226926

[18] Ager A, Boothby N, Bremer M (2009). Using the ‘protective environment’ framework to analyse children's protection needs in Darfur. Disasters.

[19] Cardoso LF (2016). What factors contribute to intimate partner violence against women in urban, conflict-affected settings? Qualitative findings from Abidjan, cote d'Ivoire. J Urban Health.

[20] Tol WA (2013). Sexual and gender-based violence in areas of armed conflict: a systematic review of mental health and psychosocial support interventions. Confl Health.

[21] Jewkes R (2002). Intimate partner violence: causes and prevention. Lancet.

[22] Gruskin S (2014). HIV and gender-based violence: welcome policies and programmes, but is the research keeping up?. Reprod Health Matters.

[23] Vyas S (2015). Exploring the association between women's access to economic resources and intimate partner violence in Dar es salaam and Mbeya, Tanzania. Soc Sci Med.

[24] Sidel VW, Levy BS (2008). The health impact of war. Int J Inj Control Saf Promot.

[25] Gupta J (2014). Associations between exposure to intimate partner violence, armed conflict, and probable PTSD among women in rural cote d'Ivoire. PLoS One.

[26] McCleary-Sills J (2016). Stigma, shame and women's limited agency in help-seeking for intimate partner violence. Glob Public Health.

[27] Wirtz AL (2013). Development of a screening tool to identify female survivors of gender-based violence in a humanitarian setting: qualitative evidence from research among refugees in Ethiopia. Confl Health.

[28] Falb KL (2016). Creating opportunities through mentorship, parental involvement, and safe spaces (COMPASS) program: multi-country study protocol to protect girls from violence in humanitarian settings. BMC Public Health.

[29] Storer HL (2016). Primary prevention is? A global perspective on how organizations engaging men in preventing gender-based violence conceptualize and Operationalize their work. Violence Against Women.

[30] Hossain M, McAlpine A (2017). Gender-based violence research methodologies in humanitarian settings: an evidence review and recommendations.

[31] Mackie G, Moneti F, Shakya H, Denny E (2012). What are social norms? How are they measured?.

[32] UNICEF (2014). Communities care: transforming lives and preventing violence toolkit.

[33] UNOCHA (2016). Humanitarian needs overview, South Sudan.

[34] UNOCHA (2016). Humanitarian needs overview, Somalia.

[35] Crabtree, B.F. and W.L. Miller. Doing qualitative research. London: Sage Publications; 1999.

[36] Alexander-Scott M, Bell E, Holden J (2016). DFID Guidence Note: Shifting Social Norms to Tackle Violence Against Women and Girls.

[37] Heise L, Manji K (2016). Social norms, GSDRC professional development reading pack.

